# Acknowledgement to Reviewers of *Children* in 2015

**DOI:** 10.3390/children3010002

**Published:** 2016-01-28

**Authors:** Children Editorial Office

**Affiliations:** MDPI AG, Klybeckstrasse 64, CH-4057 Basel, Switzerland; children@mdpi.com

The editors of *Children* would like to express their sincere gratitude to the following reviewers for assessing manuscripts in 2015.

We greatly appreciate the contribution of expert reviewers, which is crucial to the journal’s editorial decision-making process. Several steps have been taken in 2015 to thank and acknowledge reviewers. Good, timely reviews are rewarded with a discount off their next MDPI publication. By creating an account on the submission system, reviewers can access details of their past reviews, see the comments of other reviewers, and download a letter of acknowledgement for their records. This is all done, of course, within the constraints of reviewer confidentiality. Feedback from reviewers shows that most see their task as a voluntary and mostly unseen work in service to the scientific community. We are grateful to our reviewers for the contribution they make.

Alderliesten, Thomas
Allegaert, Karel
Alvan, Gunnar
Askegard-Giesmann, Johanna R.
Bernstein, Aaron
Boguski, Mark S
Booth, Josie
Bradford, Natalie
Brill, Herbert
Brough, Helen
Bublin, Merima
Büttler, Rahel
Calandri, Emanuela
Ceci, Adriana
Choonara, Imti
Cianferoni, Antonella
Devreker, Thierry
Diesner, Susanne
Doing-Harris, Kristina
Duarte, Dinah M.
Duncan, Christine N.
Edwards, Steven
Fiss, Alyssa C. LaForme
Friedl, Erika
Gigante, Joseph
Gilbert, Ruth
Gray, Peter
Hall, Alix
Hansson, Tony
Hasan, Imrul
Holland-Cunz, Stefan
Jacobs, Linda A.
Kanegaye, John Terry Zensuke
Kim, Heung Sik
Kodama, Ken
Koplin, Jennifer
Krogulska, Aneta
Lipson, Susan
Macheras, Panos
Machinena-Spera, Adrianna
Majstorovic, M.
Marr, Nico
Maslin, Kate
Megel, Mary Erickson
Mele, Cheryl
Misra, Sanghamitra M.
Mizerska-Wasiak, M
Mora, Erika
Morrison, Wynne E.
Mulcahy-Levy, Jean
Mulrooney, Daniel A.
Munywoki, Patrick K.
Muraro, Antonella
Netting, Merryn
Nguyen, Tuong-Vi
Oshiro, Bryan
Pacelli, Roberto
Pereira, Vanessa
Polack, Fernando. P.
Pole, Jason
Rosan Meyer, Rosan
Rose, Klaus
Rossie, Jean-Pierre
Schwarz, Anke
Shroba, Jodi
Sirvent, Sofía
Sluss, Dorothy
Sommer, Isolde
Stores, Gregory
Thomsen, Kelly Fair
Thornton, Sid
Troendle, David
Tuleu, Catherine
Turner, Paul J
van Driest, Sara
Veijola, Riitta
Waterston, Tony
Yu, Joyce

